# Pyrosmalite-(Fe), Fe_8_Si_6_O_15_(OH,Cl)_10_

**DOI:** 10.1107/S1600536811052822

**Published:** 2011-12-14

**Authors:** Hexiong Yang, Robert T. Downs, Yongbo W. Yang, Warren H. Allen

**Affiliations:** a Department of Geosciences, University of Arizona, 1040 E. 4th Street, Tucson, Arizona 85721-0077, USA; b Department of Chemsitry and Biochemistry, University of Arizona, 1306 E. University Blvd., Tucson, Arizona 85721-0041, USA

## Abstract

Pyrosmalite-(Fe), ideally Fe^II^_8_Si_6_O_15_(OH,Cl)_10_ [refined composition in this study: Fe_8_Si_6_O_15_(OH_0.814_Cl_0.186_)_10_·0.45H_2_O, octa­iron(II) hexa­silicate deca­(chloride/hydroxide) 0.45-hydrate], is a phyllosilicate mineral and a member of the pyrosmalite series (Fe,Mn)_8_Si_6_O_15_(OH,Cl)_10_, which includes pyrosmalite-(Mn), as well as friedelite and mcgillite, two polytypes of pyrosmalite-(Mn). This study presents the first structure determination of pyrosmalite-(Fe) based on single-crystal X-ray diffraction data from a natural sample from Burguillos del Cerro, Badajos, Spain. Pyrosmalite-(Fe) is isotypic with pyrosmalite-(Mn) and its structure is characterized by a stacking of brucite-type layers of FeO_6_-octa­hedra alternating with sheets of SiO_4_ tetra­hedra along [001]. These sheets consist of 12-, six- and four-membered rings of tetra­hedra in a 1:2:3 ratio. In contrast to previous studies on pyrosmalite-(Mn), which all assumed that Cl and one of the four OH-groups occupy the same site, our data on pyrosmalite-(Fe) revealed a split-site structure model with Cl and OH occupying distinct sites. Furthermore, our study appears to suggest the presence of disordered structural water in pyrosmalite-(Fe), consistent with infrared spectroscopic data measured from the same sample. Weak hydrogen bonding between the ordered OH-groups that are part of the brucite-type layers and the terminal silicate O atoms is present.

## Related literature

For pyrosmalite-(Fe), see: Zambonini (1901 ▶); Vaughan (1986 ▶); Pan *et al.* (1993 ▶). For other minerals of the pyrosmalite series, see: Frondel & Bauer (1953 ▶); Stillwell & McAndrew (1957 ▶); Takéuchi *et al.* (1963 ▶, 1969 ▶); Kashaev & Drits (1970 ▶); Kashaev (1968 ▶); Kato & Takéuchi (1983 ▶); Kato & Watanabe (1992 ▶); Ozawa *et al.* (1983 ▶); Abrecht (1989 ▶); Kodera *et al.* (2003 ▶). Correlations between O—H streching frequencies and O—H⋯O donor–acceptor distances were given by Libowitzky (1999 ▶). The presence of H_2_O in the pyrosmalite series was proposed by Kayupova (1964 ▶).

## Experimental

### 

#### Crystal data

Fe_8_Si_6_O_15_(OH_0.814_Cl_0.186_)_10_·0.45H_2_O
*M* *_r_* = 1067.35
Trigonal, 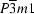

*a* = 13.3165 (2) Å
*c* = 7.0845 (2) Å
*V* = 1087.98 (4) Å^3^
*Z* = 2
Mo *K*α radiation
μ = 5.85 mm^−1^
*T* = 293 K
0.09 × 0.08 × 0.08 mm

#### Data collection

Bruker APEXII CCD area-detector diffractometer
Absorption correction: multi-scan (*SADABS*; Sheldrick, 2005 ▶) *T*_min_ = 0.622, *T*_max_ = 0.653
13410 measured reflections
1476 independent reflections
1141 reflections with *I* > 2σ(*I*)
*R*_int_ = 0.032

#### Refinement

*R*[*F* ^2^ > 2σ(*F* ^2^)] = 0.023
*wR*(*F* ^2^) = 0.068
*S* = 1.05
1476 reflections
90 parameters
All H-atom parameters refined
Δρ_max_ = 0.65 e Å^−3^
Δρ_min_ = −0.56 e Å^−3^

### 

Data collection: *APEX2* (Bruker, 2004 ▶); cell refinement: *SAINT* (Bruker, 2004 ▶); data reduction: *SAINT*; program(s) used to solve structure: *SHELXS97* (Sheldrick, 2008 ▶); program(s) used to refine structure: *SHELXL97* (Sheldrick, 2008 ▶); molecular graphics: *XtalDraw* (Downs & Hall-Wallace, 2003 ▶); software used to prepare material for publication: *publCIF* (Westrip, 2010 ▶).

## Supplementary Material

Crystal structure: contains datablock(s) I, global. DOI: 10.1107/S1600536811052822/wm2570sup1.cif

Structure factors: contains datablock(s) I. DOI: 10.1107/S1600536811052822/wm2570Isup2.hkl

Additional supplementary materials:  crystallographic information; 3D view; checkCIF report

## Figures and Tables

**Table 1:** Hydrogen-bond geometry (Å, °)

*D*—H⋯*A*	*D*—H	H⋯*A*	*D*⋯*A*	*D*—H⋯*A*
O*H*2—H1⋯O3	0.88 (4)	2.42 (4)	3.224 (2)	152 (3)
O*H*3—H2⋯O2	0.85 (4)	2.14 (4)	2.980 (2)	170 (3)
O*H*4—H3⋯O2^i^	1.03 (5)	2.66 (2)	3.247 (2)	117 (1)
O*H*4—H3⋯O2^ii^	1.03 (5)	2.66 (2)	3.247 (2)	117 (1)
O*H*4—H3⋯O2^iii^	1.03 (5)	2.66 (2)	3.247 (2)	117 (1)

Symmetry codes: (i) 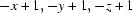; (ii) 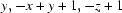; (iii) 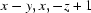.

**Fig. 1.**
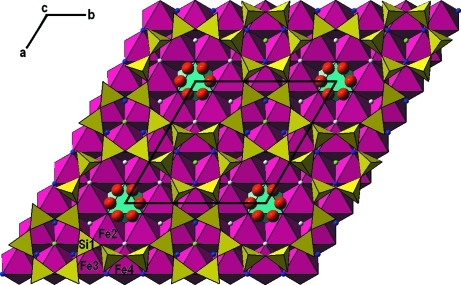
Crystal structure of pyrosmalite-(Fe). The brucite-type layers are made of Fe1(O,Cl)6 (turquoise color; site symmetry 3m.), Fe2(O,Cl)6 (.2.), Fe3O6 (.2/m), and Fe4O6 (.m.) octahedra. For clarity, the average coordinates of OH1 and Cl1 were used to draw the figure. The SiO4 tetrahedral sheets consist of 12-, 6-, and 4-membered rings with a ratio of 1:2:3. The small white, blue, and green spheres represent hydrogen atoms H1, H2, and H3, respectively. The large red spheres represent the disordered Ow1 sites that are partially occupied by H2O molecules.

**Fig. 2.**
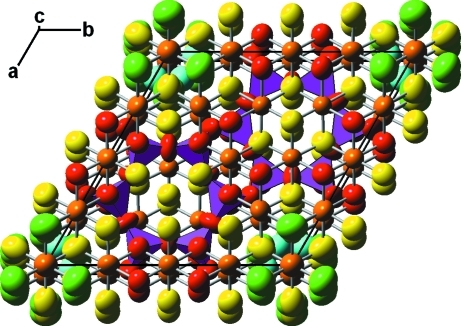
Atoms in pyrosmalite-(Fe) with corresponding ellipsoids at thw 99% probability level. For clarity, the SiO4 groups are shown as purple tetrahedra. Brown, red, yellow, and green ellipsoids represent Fe, O, OH, and Cl atoms, respectively. Turquoise spheres represent H2O. Hydrogen atoms cannot be seen from this direction.

**Fig. 3.**
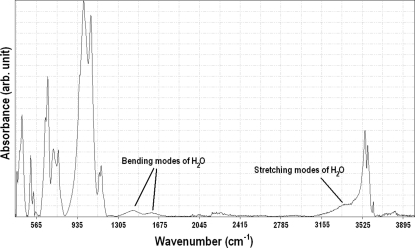
Infrared spectrum of pyrosmalite-(Fe).

## supplementary crystallographic information

### Comment

Pyrosmalite is the name given to the phyllosilicate series with the general chemical formula (Fe,Mn)_8_Si_6_O_15_(OH,Cl)_10_. Minerals of the pyrosmalite series are generally related to metamorphism in close association with Fe- and Mn-rich silicates and oxides (*e.g.*, Frondel & Bauer, 1953; Stillwell & McAndrew, 1957; Vaughan, 1986; Abrecht, 1989; Pan *et al.*, 1993; Kodera *et al.*, 2003). The Fe-rich members of the series are called pyrosmalite-(Fe) (previously ferropyrosmalite), whereas the Mn-rich members include pyrosmalite-(Mn) (previously manganpyrosmalite), and friedelite, a polytype of the series with the *c*-axis three times that of pyrosmalite-(Mn), as well as mcgillite, Mn_8_Si_6_O_15_(OH)_8_Cl_2_, an ordered form of friedelite with the *c*-axis twelve times that of pyrosmalite-(Mn) (Ozawa *et al.*, 1983). The polytypism in the pyrosmalite group of minerals has been regarded to be similar to that of the micas (Frondel & Bauer, 1953; Takéuchi *et al.*, 1969; Kashaev & Drits, 1970; Kato & Takéuchi, 1983; Ozawa *et al.*, 1983).

The crystal structure of pyrosmalite-(Mn) was first investigated by Takéuchi *et al.* (1963) without giving any detailed structure information. Kashaev (1968) reported a partial structure model for pyrosmalite-(Mn) based on photographic X-ray intensity data of 35 reflections collected from a crystal with *X*_Fe_ = Fe / (Fe + Mn) = 0.39. By means of Weissenberg and precession methods, Takéuchi *et al.* (1969) determined the structure of pyrosmalite-(Mn) from a crystal with *X*_Fe_ = 0.18 (*R* = 19.8%). Using a four-circle X-ray diffractometer, Kato & Takéuchi (1983) examined two pyrosmalite-(Mn) crystals, one having *X*_Fe_ = 0.46 and the other *X*_Fe_ = 0.18. Their structure refinements on atomic coordinates and isotropic displacement parameters resulted in *R* = 6.0% and 10.5% for the former and latter crystals, respectively. The structure of friedelite was solved by Kato & Watanabe (1992) in space group *C*2/*m* (*R* = 20.3%). However, despite its first description in the early twentieth century (Zambonini, 1901), the structure of pyrosmalite-(Fe) has remained undetermined hitherto. This study presents the first structure refinement of pyrosmalite-(Fe) on the basis of single-crystal X-ray diffraction data.

Pyrosmalite-(Fe) is isotypic with pyrosmalite-(Mn) (Kashaev, 1968; Takéuchi *et al.*, 1969; Kato & Takéuchi, 1983). Its structure is characterized by brucite-type layers of FeO_6_-octahedra alternating with sheets of SiO_4_ tetrahedra along [001]. The tetrahedral sheets consist of 12-, 6-, and 4-membered rings of SiO_4_ tetrahedra with a ratio of 1:2:3 (Figs. 1 and 2). Kato & Takéuchi (1983) noted that the SiO_4_ tetrahedra in pyrosmalite-(Mn) are elongated towards their apical oxygen atoms (O4). Similar results have also been found in pyrosmalite-(Fe). The average length (2.678 Å) of the pyramidal edges is 4.3% longer than that (2.568 Å) of the basal edges. It is intriguing to note that all previous structure refinements on pyrosmalite-(Mn) assumed a disordered model with Cl and OH1 occupying the same site, which resulted in a markedly large isotropic displacement parameter for the site that is more than twice as large as that of other anion sites, and *R* > 6% (Takéuchi *et al.*, 1969; Kato & Takéuchi, 1983). Using the same disorder model for Cl and OH1, we arrived at similar results [*R*_1_ = 6.1%, GOF = 1.621, and an unreasonably large *U*_iso_ value for the (OH1,Cl) site]. Examination of difference Fourier maps from our structure refinements, nonetheless, uncovered an outstanding residual peak that is 0.74 Å away from OH1. By introducing a split-site model, in which Cl and OH1 occupy symmetrically distinct sites, we obtained *R*_1_ = 2.89% and GOF = 1.076. The refined Cl content from the split-site model is 1.86 atoms per formula unit (apfu), in excellent agreement with the value of 1.7 apfu from the electron microprobe analysis.

Another interesting feature from our structure refinement on pyrosmalite-(Fe) is a small, but noticeable residual peak in the difference Fourier synthesis, which is located within the 12-membered tetrahedral rings (Fig. 1). Because the determined structure formula is charge-balanced without considering this site, the best assignment for the site would be a disordered water molecule. With this assumption, a further refinement reduced *R*_1_ from 2.89 to 2.32%, which yielded 15% site occupancy of H_2_O, or an overall structure formula (Fe,Mn)_8_Si_6_O_15_(OH_0.814_Cl_0.186_)_10_^.^0.45H_2_O. The detection of the existence of H_2_O in pyrosmalite-(Fe) appears to be consistent with our infrared spectral measurement on the same sample studied (Fig. 3) (http://rruff.info/R050158). Specifically, the two weak, broad bands at 1450 and 1613 cm^-1^ can be attributed to the bending modes of H_2_O and the broad shoulder at ~3367 cm^-1^ to the stretching mode of H_2_O. Additionally, three relatively sharp bands at 3550, 3574, and 3625 cm^-1^ may be assigned to the O—H stretching modes related to three weak hydrogen bonds OH3···O2, OH2···O3, and OH4···O2, respectively, according to the correlation between O—H stretching frequencies and O—H···O hydrogen bond lengths (Libowitzky, 1999). In fact, the presence of H_2_O in the pyrosmalite series has been proposed by Kayupova (1964), who presented a chemical formula of (Mn,Fe,Zn)_8_Si_6_O_15_(OH,Cl)_10_^.^1.1H_2_O for pyrosmalite-(Mn) from the Broken Hill deposit, Australia, and (Mn,Fe)_8_Si_6_O_15_(OH,Cl)_10_^.^1.5H_2_O for pyrosmalite-(Mn) from the Ushkatyn I deposit, Kazakhstan. Accordingly, our structure determination on pyrosmalite-(Fe) requires more systematic and detailed investigations on the possible existence of structural water in other minerals of the pyrosmalite series.

### Experimental

The pyrosmalite-(Fe) crystal used in this study is from Burguillos del Cerro, Badajos, Spain and is in the collection of the RRUFF project (deposition No. R050158; http://rruff.info). The empirical chemical formula, (Fe^2+^_0.92_Mn^2+^_0.06_Mg_0.02_)_8_Si_6_O_15_(OH_0.83_Cl_0.17_)_10_, was determined with a CAMECA SX50 ele ctron microprobe at the conditions of 15 kV, 20 nA, and a beam size of 10 µm (http//rruff.info).

### Refinement

Three H-atoms were located near OH2, OH3, and OH4 from difference Fourier syntheses and their positions refined freely with a fixed isotropic displacement parameter (*U_iso_* = 0.03). The Ow1 site, partially occupied by H_2_O, was refined with the isotropic displacement parameter only. During the structure refinements, the small amount of Mn was treated as Fe, because of their similar X-ray scattering powers. In addition, the refinement assumed full occupancy of all octahedral sites by Fe, as the overall effects of the trace amount of Mg on the final structure results are negligible. The highest residual peak in the difference Fourier maps was located at (0.3388, 0.4390, 0.2383), 0.67 Å from O4, and the deepest hole at (0.5198, 0.4802, 0.9750), 0.49 Å from Fe3.

### Crystal data

**Table:**

Fe_8_Si_6_O_15_(OH_0.814_Cl_0.186_)_10_·0.45H_2_O	*D*_x_ = 3.253 Mg m^−^^3^
*M**_r_* = 1067.35	Mo *K*α radiation, λ = 0.71073 Å
Trigonal, *P*3*m*1	Cell parameters from 2724 reflections
Hall symbol: -P 3 2"	θ = 2.9–32.6°
*a* = 13.3165 (2) Å	µ = 5.85 mm^−^^1^
*c* = 7.0845 (2) Å	*T* = 293 K
*V* = 1087.98 (4) Å^3^	Cuboid, light green
*Z* = 2	0.09 × 0.08 × 0.08 mm
*F*(000) = 1039	

### Data collection

**Table:**

Bruker APEXII CCD area-detector diffractometer	1476 independent reflections
Radiation source: fine-focus sealed tube	1141 reflections with *I* > 2σ(*I*)
graphite	*R*_int_ = 0.032
φ and ω scan	θ_max_ = 32.6°, θ_min_ = 1.8°
Absorption correction: multi-scan (*SADABS*; Sheldrick, 2005)	*h* = −18→20
*T*_min_ = 0.622, *T*_max_ = 0.653	*k* = −20→16
13410 measured reflections	*l* = −10→10

### Refinement

**Table:**

Refinement on *F*^2^	Secondary atom site location: difference Fourier map
Least-squares matrix: full	Hydrogen site location: difference Fourier map
*R*[*F*^2^ > 2σ(*F*^2^)] = 0.023	All H-atom parameters refined
*wR*(*F*^2^) = 0.068	*w* = 1/[σ^2^(*F*_o_^2^) + (0.0328*P*)^2^ + 0.4308*P*] where *P* = (*F*_o_^2^ + 2*F*_c_^2^)/3
*S* = 1.05	(Δ/σ)_max_ = 0.002
1476 reflections	Δρ_max_ = 0.65 e Å^−^^3^
90 parameters	Δρ_min_ = −0.56 e Å^−^^3^
0 restraints	Extinction correction: *SHELXL97* (Sheldrick, 2008), Fc^*^=kFc[1+0.001xFc^2^λ^3^/sin(2θ)]^-1/4^
Primary atom site location: structure-invariant direct methods	Extinction coefficient: 0.00082 (13)

### Special details

**Table:**

Geometry. All e.s.d.'s (except the e.s.d. in the dihedral angle between two l.s. planes) are estimated using the full covariance matrix. The cell e.s.d.'s are taken into account individually in the estimation of e.s.d.'s in distances, angles and torsion angles; correlations between e.s.d.'s in cell parameters are only used when they are defined by crystal symmetry. An approximate (isotropic) treatment of cell e.s.d.'s is used for estimating e.s.d.'s involving l.s. planes.
Refinement. Refinement of *F*^2^ against ALL reflections. The weighted *R*-factor *wR* and goodness of fit *S* are based on *F*^2^, conventional *R*-factors *R* are based on *F*, with *F* set to zero for negative *F*^2^. The threshold expression of *F*^2^ > σ(*F*^2^) is used only for calculating *R*-factors(gt) *etc*. and is not relevant to the choice of reflections for refinement. *R*-factors based on *F*^2^ are statistically about twice as large as those based on *F*, and *R*- factors based on ALL data will be even larger.

### Fractional atomic coordinates and isotropic or equivalent isotropic displacement parameters (Å^2^)

**Table:**

	*x*	*y*	*z*	*U*_iso_*/*U*_eq_	Occ. (<1)
Fe1	0.0000	0.0000	0.0000	0.01547 (19)	
Fe2	0.25510 (3)	0.0000	0.0000	0.01193 (10)	
Fe3	0.5000	0.0000	0.0000	0.01004 (12)	
Fe4	0.50261 (3)	0.251306 (15)	0.01962 (5)	0.00953 (10)	
Si1	0.43696 (4)	0.10405 (4)	0.62679 (6)	0.00674 (11)	
O1	0.34125 (13)	0.0000	0.5000	0.0126 (3)	
O2	0.56373 (8)	0.12746 (15)	0.5610 (2)	0.0113 (3)	
O3	0.43070 (15)	0.21535 (8)	0.5580 (2)	0.0122 (3)	
O4	0.41964 (10)	0.08283 (10)	0.84971 (18)	0.0100 (3)	
Cl1	0.16942 (9)	0.08471 (4)	0.7741 (2)	0.0189 (4)	0.619 (5)
OH1	0.1647 (4)	0.0824 (2)	0.8785 (9)	0.0133 (11)	0.381 (5)
OH2	0.33476 (14)	0.16738 (7)	0.1306 (2)	0.0129 (4)	
OH3	0.58147 (7)	0.16295 (14)	0.1443 (2)	0.0106 (3)	
OH4	0.3333	0.6667	0.1263 (4)	0.0111 (6)	
OW1	0.1055 (17)	0.1055 (17)	0.5000	0.069 (8)*	0.147 (8)
H1	0.334 (3)	0.1671 (14)	0.255 (5)	0.030*	
H2	0.5810 (14)	0.162 (3)	0.264 (6)	0.030*	
H3	0.3333	0.6667	0.271 (8)	0.030*	

### Atomic displacement parameters (Å^2^)

**Table:**

	*U*^11^	*U*^22^	*U*^33^	*U*^12^	*U*^13^	*U*^23^
Fe1	0.0118 (3)	0.0118 (3)	0.0229 (4)	0.00589 (14)	0.000	0.000
Fe2	0.01001 (15)	0.00849 (18)	0.01679 (17)	0.00424 (9)	0.00094 (6)	0.00188 (11)
Fe3	0.00893 (19)	0.0089 (2)	0.0123 (2)	0.00444 (12)	0.00070 (7)	0.00141 (15)
Fe4	0.00747 (18)	0.00783 (14)	0.01318 (15)	0.00373 (9)	0.00033 (10)	0.00016 (5)
Si1	0.0067 (2)	0.0056 (2)	0.00817 (17)	0.00325 (17)	−0.00031 (14)	−0.00047 (14)
O1	0.0101 (6)	0.0098 (9)	0.0178 (7)	0.0049 (4)	−0.0028 (3)	−0.0056 (6)
O2	0.0089 (6)	0.0146 (9)	0.0122 (6)	0.0073 (4)	0.0002 (3)	0.0005 (6)
O3	0.0176 (9)	0.0094 (6)	0.0124 (6)	0.0088 (5)	−0.0015 (6)	−0.0008 (3)
O4	0.0105 (6)	0.0116 (6)	0.0084 (5)	0.0060 (5)	0.0004 (4)	0.0013 (4)
Cl1	0.0153 (6)	0.0195 (5)	0.0205 (8)	0.0076 (3)	−0.0003 (4)	−0.00016 (18)
OH1	0.018 (3)	0.0133 (19)	0.010 (3)	0.0090 (14)	−0.0013 (16)	−0.0007 (8)
OH2	0.0128 (9)	0.0130 (7)	0.0130 (7)	0.0064 (4)	0.0030 (6)	0.0015 (3)
OH3	0.0114 (6)	0.0127 (9)	0.0082 (6)	0.0063 (4)	0.0006 (3)	0.0013 (5)
OH4	0.0109 (9)	0.0109 (9)	0.0117 (11)	0.0054 (5)	0.000	0.000

### Geometric parameters (Å, °)

**Table:**

Fe1—OH1^i^	2.085 (5)	Fe2—Cl1^iv^	2.5342 (10)
Fe1—OH1^ii^	2.085 (5)	Fe2—Cl1^iii^	2.5342 (10)
Fe1—OH1^iii^	2.085 (5)	Fe3—OH3^ix^	2.1394 (16)
Fe1—OH1^iv^	2.085 (5)	Fe3—OH3	2.1394 (16)
Fe1—OH1^v^	2.085 (5)	Fe3—O4^iv^	2.1624 (11)
Fe1—OH1^vi^	2.085 (5)	Fe3—O4^x^	2.1624 (11)
Fe1—Cl1^ii^	2.5255 (12)	Fe3—O4^viii^	2.1624 (11)
Fe1—Cl1^i^	2.5255 (12)	Fe3—O4^xi^	2.1624 (11)
Fe1—Cl1^vi^	2.5255 (12)	Fe4—OH2	2.0893 (17)
Fe1—Cl1^iii^	2.5255 (12)	Fe4—OH3^xii^	2.1222 (10)
Fe1—Cl1^iv^	2.5255 (12)	Fe4—OH3	2.1222 (10)
Fe1—Cl1^v^	2.5255 (12)	Fe4—OH4^xiii^	2.1560 (13)
Fe2—OH2	2.1413 (11)	Fe4—O4^iv^	2.2856 (12)
Fe2—OH2^vii^	2.1413 (11)	Fe4—O4^xiv^	2.2856 (12)
Fe2—OH1^iv^	2.171 (3)	Si1—O4	1.6006 (13)
Fe2—OH1^iii^	2.171 (3)	Si1—O3	1.6014 (6)
Fe2—O4^iv^	2.1759 (12)	Si1—O1	1.6078 (8)
Fe2—O4^viii^	2.1759 (11)	Si1—O2	1.6242 (7)
			
OH1^i^—Fe1—OH1^ii^	180.0 (4)	OH2—Fe2—OH1^iv^	75.92 (12)
OH1^i^—Fe1—OH1^iii^	104.15 (19)	OH2^vii^—Fe2—OH1^iv^	101.70 (12)
OH1^ii^—Fe1—OH1^iii^	75.85 (19)	OH2—Fe2—OH1^iii^	101.70 (12)
OH1^i^—Fe1—OH1^iv^	75.85 (19)	OH2^vii^—Fe2—OH1^iii^	75.92 (12)
OH1^ii^—Fe1—OH1^iv^	104.15 (19)	OH1^iv^—Fe2—OH1^iii^	72.4 (3)
OH1^iii^—Fe1—OH1^iv^	75.85 (19)	OH2—Fe2—O4^iv^	80.48 (6)
OH1^i^—Fe1—OH1^v^	75.85 (19)	OH2^vii^—Fe2—O4^iv^	101.72 (5)
OH1^ii^—Fe1—OH1^v^	104.15 (19)	OH1^iv^—Fe2—O4^iv^	102.83 (15)
OH1^iii^—Fe1—OH1^v^	180.00 (18)	OH1^iii^—Fe2—O4^iv^	173.86 (16)
OH1^iv^—Fe1—OH1^v^	104.15 (19)	OH2—Fe2—O4^viii^	101.72 (5)
OH1^i^—Fe1—OH1^vi^	104.15 (19)	OH2^vii^—Fe2—O4^viii^	80.48 (6)
OH1^ii^—Fe1—OH1^vi^	75.85 (19)	OH1^iv^—Fe2—O4^viii^	173.86 (16)
OH1^iii^—Fe1—OH1^vi^	104.15 (19)	OH1^iii^—Fe2—O4^viii^	102.83 (15)
OH1^iv^—Fe1—OH1^vi^	180.0 (4)	O4^iv^—Fe2—O4^viii^	82.21 (6)
OH1^v^—Fe1—OH1^vi^	75.85 (19)	OH2—Fe2—Cl1^iv^	84.75 (4)
OH1^i^—Fe1—Cl1^ii^	165.06 (15)	OH2^vii^—Fe2—Cl1^iv^	93.31 (5)
OH1^ii^—Fe1—Cl1^ii^	14.94 (15)	OH1^iv^—Fe2—Cl1^iv^	15.84 (15)
OH1^iii^—Fe1—Cl1^ii^	84.79 (13)	OH1^iii^—Fe2—Cl1^iv^	82.88 (16)
OH1^iv^—Fe1—Cl1^ii^	95.21 (13)	O4^iv^—Fe2—Cl1^iv^	91.65 (4)
OH1^v^—Fe1—Cl1^ii^	95.21 (13)	O4^viii^—Fe2—Cl1^iv^	170.13 (4)
OH1^vi^—Fe1—Cl1^ii^	84.79 (13)	OH2—Fe2—Cl1^iii^	93.31 (5)
OH1^i^—Fe1—Cl1^i^	14.94 (15)	OH2^vii^—Fe2—Cl1^iii^	84.75 (4)
OH1^ii^—Fe1—Cl1^i^	165.06 (15)	OH1^iv^—Fe2—Cl1^iii^	82.88 (16)
OH1^iii^—Fe1—Cl1^i^	95.21 (13)	OH1^iii^—Fe2—Cl1^iii^	15.84 (15)
OH1^iv^—Fe1—Cl1^i^	84.79 (13)	O4^iv^—Fe2—Cl1^iii^	170.13 (4)
OH1^v^—Fe1—Cl1^i^	84.79 (13)	O4^viii^—Fe2—Cl1^iii^	91.65 (4)
OH1^vi^—Fe1—Cl1^i^	95.21 (13)	Cl1^iv^—Fe2—Cl1^iii^	95.43 (6)
Cl1^ii^—Fe1—Cl1^i^	180.00 (7)	OH3^ix^—Fe3—OH3	180.00 (8)
OH1^i^—Fe1—Cl1^vi^	95.21 (13)	OH3^ix^—Fe3—O4^iv^	98.78 (4)
OH1^ii^—Fe1—Cl1^vi^	84.79 (13)	OH3—Fe3—O4^iv^	81.22 (4)
OH1^iii^—Fe1—Cl1^vi^	95.21 (13)	OH3^ix^—Fe3—O4^x^	81.22 (4)
OH1^iv^—Fe1—Cl1^vi^	165.06 (15)	OH3—Fe3—O4^x^	98.78 (4)
OH1^v^—Fe1—Cl1^vi^	84.79 (13)	O4^iv^—Fe3—O4^x^	180.00 (7)
OH1^vi^—Fe1—Cl1^vi^	14.94 (15)	OH3^ix^—Fe3—O4^viii^	81.22 (4)
Cl1^ii^—Fe1—Cl1^vi^	95.87 (4)	OH3—Fe3—O4^viii^	98.78 (4)
Cl1^i^—Fe1—Cl1^vi^	84.13 (4)	O4^iv^—Fe3—O4^viii^	82.83 (6)
OH1^i^—Fe1—Cl1^iii^	95.21 (13)	O4^x^—Fe3—O4^viii^	97.17 (6)
OH1^ii^—Fe1—Cl1^iii^	84.79 (13)	OH3^ix^—Fe3—O4^xi^	98.78 (4)
OH1^iii^—Fe1—Cl1^iii^	14.94 (15)	OH3—Fe3—O4^xi^	81.22 (4)
OH1^iv^—Fe1—Cl1^iii^	84.79 (13)	O4^iv^—Fe3—O4^xi^	97.17 (6)
OH1^v^—Fe1—Cl1^iii^	165.06 (15)	O4^x^—Fe3—O4^xi^	82.83 (6)
OH1^vi^—Fe1—Cl1^iii^	95.21 (13)	O4^viii^—Fe3—O4^xi^	180.00 (7)
Cl1^ii^—Fe1—Cl1^iii^	95.87 (4)	OH2—Fe4—OH3^xii^	103.91 (5)
Cl1^i^—Fe1—Cl1^iii^	84.13 (4)	OH2—Fe4—OH3	103.91 (5)
Cl1^vi^—Fe1—Cl1^iii^	84.13 (4)	OH3^xii^—Fe4—OH3	106.62 (9)
OH1^i^—Fe1—Cl1^iv^	84.79 (13)	OH2—Fe4—OH4^xiii^	173.45 (7)
OH1^ii^—Fe1—Cl1^iv^	95.21 (13)	OH3^xii^—Fe4—OH4^xiii^	79.84 (5)
OH1^iii^—Fe1—Cl1^iv^	84.79 (13)	OH3—Fe4—OH4^xiii^	79.84 (5)
OH1^iv^—Fe1—Cl1^iv^	14.94 (15)	OH2—Fe4—O4^iv^	79.07 (5)
OH1^v^—Fe1—Cl1^iv^	95.21 (13)	OH3^xii^—Fe4—O4^iv^	172.72 (5)
OH1^vi^—Fe1—Cl1^iv^	165.06 (15)	OH3—Fe4—O4^iv^	78.78 (5)
Cl1^ii^—Fe1—Cl1^iv^	84.13 (4)	OH4^xiii^—Fe4—O4^iv^	96.59 (5)
Cl1^i^—Fe1—Cl1^iv^	95.87 (4)	OH2—Fe4—O4^xiv^	79.07 (5)
Cl1^vi^—Fe1—Cl1^iv^	180.00 (7)	OH3^xii^—Fe4—O4^xiv^	78.78 (5)
Cl1^iii^—Fe1—Cl1^iv^	95.87 (4)	OH3—Fe4—O4^xiv^	172.72 (5)
OH1^i^—Fe1—Cl1^v^	84.79 (13)	OH4^xiii^—Fe4—O4^xiv^	96.59 (5)
OH1^ii^—Fe1—Cl1^v^	95.21 (13)	O4^iv^—Fe4—O4^xiv^	95.44 (6)
OH1^iii^—Fe1—Cl1^v^	165.06 (15)	O4—Si1—O3	113.19 (7)
OH1^iv^—Fe1—Cl1^v^	95.21 (13)	O4—Si1—O1	114.64 (5)
OH1^v^—Fe1—Cl1^v^	14.94 (15)	O3—Si1—O1	104.01 (7)
OH1^vi^—Fe1—Cl1^v^	84.79 (13)	O4—Si1—O2	111.17 (7)
Cl1^ii^—Fe1—Cl1^v^	84.13 (4)	O3—Si1—O2	105.38 (9)
Cl1^i^—Fe1—Cl1^v^	95.87 (4)	O1—Si1—O2	107.77 (8)
Cl1^vi^—Fe1—Cl1^v^	95.87 (4)	Si1—O1—Si1^viii^	137.58 (12)
Cl1^iii^—Fe1—Cl1^v^	180.00 (4)	Si1—O2—Si1^xv^	141.27 (11)
Cl1^iv^—Fe1—Cl1^v^	84.13 (4)	Si1^xvi^—O3—Si1	144.19 (10)
OH2—Fe2—OH2^vii^	177.13 (8)		

Symmetry codes: (i) *x*−*y*, *x*, −*z*+1; (ii) −*x*+*y*, −*x*, *z*−1; (iii) *y*, −*x*+*y*, −*z*+1; (iv) *x*, *y*, *z*−1; (v) −*y*, *x*−*y*, *z*−1; (vi) −*x*, −*y*, −*z*+1; (vii) *y*, −*x*+*y*, −*z*; (viii) *x*−*y*, −*y*, −*z*+1; (ix) −*x*+1, −*y*, −*z*; (x) −*x*+1, −*y*, −*z*+1; (xi) −*x*+*y*+1, *y*, *z*−1; (xii) −*x*+*y*+1, −*x*+1, *z*; (xiii) −*x*+1, −*y*+1, −*z*; (xiv) *x*, *x*−*y*, *z*−1; (xv) −*x*+*y*+1, *y*, *z*; (xvi) *x*, *x*−*y*, *z*.

### Hydrogen-bond geometry (Å, °)

**Table:**

*D*—H···*A*	*D*—H	H···*A*	*D*···*A*	*D*—H···*A*
OH2—H1···O3	0.88 (4)	2.42 (4)	3.224 (2)	152 (3)
OH3—H2···O2	0.85 (4)	2.14 (4)	2.980 (2)	170 (3)
OH4—H3···O2^xvii^	1.03 (5)	2.66 (2)	3.247 (2)	117.(1)
OH4—H3···O2^xviii^	1.03 (5)	2.66 (2)	3.247 (2)	117.(1)
OH4—H3···O2^i^	1.03 (5)	2.66 (2)	3.247 (2)	117.(1)

Symmetry codes: (xvii) −*x*+1, −*y*+1, −*z*+1; (xviii) *y*, −*x*+*y*+1, −*z*+1; (i) *x*−*y*, *x*, −*z*+1.
